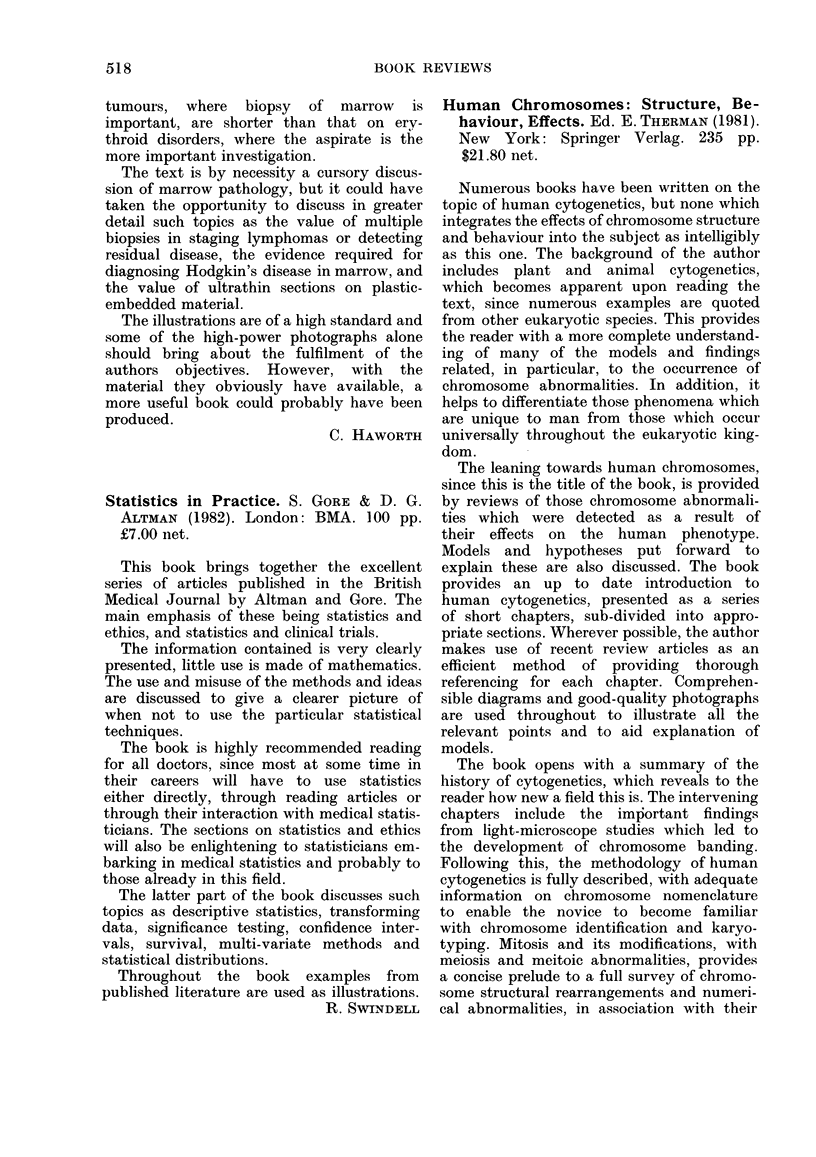# Statistics in Practice

**Published:** 1982-09

**Authors:** R. Swindell


**Statistics in Practice.** S. GORE & D. G. ALTMAN (1982). London: BMA. 100 pp. £7.00 net.

This book brings together the excellent series of articles published in the British Medical Journal by Altman and Gore. The main emphasis of these being statistics and ethics, and statistics and clinical trials.

The information contained is very clearly presented, little use is made of mathematics. The use and misuse of the methods and ideas are discussed to give a clearer picture of when not to use the particular statistical techniques.

The book is highly recommended reading for all doctors, since most at some time in their careers will have to use statistics either directly, through reading articles or through their interaction with medical statisticians. The sections on statistics and ethics will also be enlightening to statisticians embarking in medical statistics and probably to those already in this field.

The latter part of the book discusses such topics as descriptive statistics, transforming data, significance testing, confidence intervals, survival, multi-variate methods and statistical distributions.

Throughout the book examples from published literature are used as illustrations.

R. SWINDELL